# Chronic postsurgical inguinal pain: incidence and diagnostic biomarkers from a large German national claims database

**DOI:** 10.1016/j.bja.2024.11.048

**Published:** 2025-02-04

**Authors:** Eva Herrmann, Magnus Schindehütte, Gudrun Kindl, Ann-Kristin Reinhold, Felix Aulbach, Norman Rose, Johannes Dreiling, Daniel Schwarzkopf, Michael Meir, Yuying Jin, Karolin Teichmüller, Anna Widder, Robert Blum, Abdelrahman Sawalma, Nadine Cebulla, Michael Sendtner, Winfried Meissner, Alexander Brack, Mirko Pham, Claudia Sommer, Nicolas Schlegel, Heike L. Rittner

**Affiliations:** 1 Department of Anaesthesiology, Intensive Care, Emergency and Pain Medicine, Centre for Interdisciplinary Pain Medicine, University Hospital Würzburg, Würzburg, Germany; 2 Department of Neuroradiology, University Hospital Würzburg, Würzburg, Germany; 3 Department of Anaesthesiology and Intensive Care Medicine, Section Pain Therapy, University Hospital Jena, Jena, Germany; 4 Department of Surgery I, University Hospital Würzburg, Würzburg, Germany; 5 Department of Neurology, University Hospital Würzburg, Würzburg, Germany; 6 Institute for Neurobiology, University Hospital Würzburg, Würzburg, Germany

**Keywords:** chronic postsurgical pain, cytokines, dorsal root ganglion, hernia surgery, imaging, lipids

## Abstract

**Background:**

Chronic postsurgical inguinal pain (CPIP) is the most common complication of groin hernia surgery. The characteristics of patients, their medical care, and choice of diagnostic tools remain to be defined to optimise preventive and therapeutic interventions.

**Methods:**

Claims data from 2018 and a 1-yr follow-up were analysed for incidence and medical care. A separate cohort (141 healthy controls and 17 CPIP patients) was examined by deep phenotyping. This included sensory testing, blood and skin biopsies, MRI of the dorsal root ganglion (DRG), and patient-reported outcomes.

**Results:**

Of 11,221 patients with hernia surgery in 2018 identified, 8.5% had pain before that was relieved by surgery, but a similar percentage had novel pain in this region. Deep phenotyping of 141 healthy controls provided a map of the inguinal sensory system. The following analysis of patients with CPIP revealed that they suffered from moderate pain with neuropathic features, individual sensory abnormalities, and unilateral L1 DRG atrophy. In the blood, levels of C-C-motif chemokine ligand (CCL2) and brain-derived neurotrophic factor (BDNF) were upregulated, whereas apolipoprotein A1 (ApoA1) concentration was reduced. A cluster of DRG atrophy, BDNF, ApoA1, and anxiety correlated best with the diagnosis. CPIP patients with novel pain had significantly more DRG atrophy (–24% ipsilateral *vs* contralateral volume).

**Conclusions:**

CPIP is often newly acquired after surgery. A combination of DRG imaging, serum markers, and anxiety screening can support the diagnosis. In the future, this could guide clinicians towards more personalised therapies (e.g. targeting anxiety or lipid profiles) and possible altered surgical techniques.

**Clinical trial registration:**

German Trial Registry DRKS00024588 and DRKS00016790.

**Editor's key points**

• Chronic postsurgical inguinal pain (CPIP) is a common form of chronic postsurgical pain after groin hernia surgery, but its characteristic diagnostic markers are poorly defined.
• Of 11,221 patients undergoing hernia surgery, 8.5% had pain before that was relieved by surgery, and a similar percentage had new-onset groin pain.
• Deep phenotyping showed increased levels of the cytokine CCL2 and brain-derived neurotrophic factor, reduced apolipoprotein A1 and ipsilateral dorsal root ganglion atrophy, and with increased anxiety were associated with CPIP.
• These findings identify additional potential diagnostic tools and therapeutic targets for future investigation.

With more than 20 million operations annually, groin hernia repair is one of the most common surgical procedures worldwide.^1^ The development of chronic postsurgical inguinal pain (CPIP) is the most frequent problem in these patients with an incidence of 10–54% depending on the criteria applied for the definition of CPIP.^2^ Despite technical advances in surgery, the incidence of CPIP has not declined in recent decades.^3^^,^^4^

Chronic postsurgical pain develops or increases in intensity beyond the wound healing process ≥3 months after a surgical procedure.^3^ It is localised in the surgical area or its projection zones and often shows neuropathic pain characteristics. Known risk factors for chronic postsurgical pain include high levels of presurgical pain, severe postoperative pain, and psychological and other individual patient-related factors.^3^^,^^5^ The pathophysiology of CPIP is not clear; 31–38% of patients have a neuropathic component,^6^^,^^7^ which is considered to be maintained by local and systemic inflammation.^8^^,^^9^ A causative role of inflammation in the onset of CPIP has been assumed considering data from a small trial using antitumour necrosis factor-alpha (TNF-α) antibodies, which led to reduced pain.^10^

All sensory stimuli of the groin are processed via the paired dorsal root ganglia (DRG) in the spinal cord segment L1. Whether local or systemic inflammatory changes, alterations in intraepidermal nerve fibre density (IENFD) at the site of surgery, or changes in the DRG itself occur in patients with CPIP remains unclear. The diagnosis of CPIP depends on clinical parameters that are subjective in nature. Accordingly, appropriate patient-tailored therapeutic approaches based on the pathophysiology or diagnostic tools of the CPIP phenotype are missing.

The present study was conducted to first define the relevance of CPIP and its treatment in real-world data. Because of the lack of normative data for possible neuropathy in CPIP, deep phenotyping of the groin in healthy controls was done as the second step. Finally, we used this combined knowledge to study the pathophysiology of CPIP and evaluate possible neuropathy in a separate cohort of patients affected by this condition.

## Methods

A detailed description of the methods is found in Supplementary material.

### Analysis of healthcare data for the insurance cohort

We used anonymised data from the scientific data warehouse of the German health insurance (BARMER GEK, DRKS00024588) as part of the LOPSTER project. Using the Operationen- und Prozedurenschlüssel (OPS) classification of procedures (OPS codes 5-530 or 5-531), we identified patients who underwent hernia surgery in 2018**.** As CPIP is not coded in the ICD-10, surrogate diagnoses, specifically R10.2–4 (pelvic and perineal pain; pain with localisation in other parts of the lower abdomen; other and unspecified abdominal pain) and M79.25 (neuralgia and neuritis, unspecified: pelvic region/thigh) were used before and after surgery. We therefore defined pain after surgery in this region as ‘probable CPIP’ and referred to these individuals as the ‘insurance cohort’. Sociodemographic and healthcare parameters were obtained 1 yr before and after the index year 2018. Medication, nonpharmacological therapies, and psychiatric comorbidities were obtained for up to 12 months after surgery (Supplementary Fig. S1).

### Assessment of the healthy control cohort

The exploratory cross-sectional unicentre *ResolvePAIN* study protocol was approved (DRKS00016790). The healthy control group comprised 141 cases subdivided into three age groups based on a sample size calculation using variability of pressure pain thresholds based on age and sex (Supplementary Table S1).^11^ All study participants underwent clinical assessment, patient-reported outcomes for pain and comorbidities, and quantitative sensory testing (QST) of the groin and hand for comparison. If they gave consent, an inguinal skin biopsy was performed (Supplementary Fig. S1).

### Assessment of the pain cohort

Seventeen patients with CPIP diagnosed according to IASP^12^ and the Hernia Surgery Group^13^ were recruited from the outpatient clinic of the Centre for Interdisciplinary Pain Medicine and the Department of Surgery, and newspaper advertisements.^5^ These patients formed the ‘pain cohort’. Pain before surgery was assessed by telephone interview up to 1–4 yr after the initial assessment. Subjects in the pain cohort underwent the same battery of tests as the healthy control cohort stated above. In addition, an MRI of the groin and DRGs was performed (Supplementary Fig. S1).

### Building a model to identify measures associated with chronic postsurgical inguinal pain

A model was developed to identify relevant measures associated with CPIP using machine learning techniques, as detailed in the Supplemental material. In short, the model construction followed a two-step approach.^14^, ^15^ Initially, a random forest algorithm was used to calculate the mean decrease impurity (MDI), which serves as a measure of feature importance. The MDI analysis identified four key variables (brain-derived neurotrophic factor [BDNF], apolipoprotein A1 [ApoA1], C-C-motif chemokine ligand [CCL2], and STAI-T) as important for distinguishing between CPIP patients of the pain cohort and control cohort. These variables were subsequently included in a binary logistic regression model, with the diagnosis (CPIP *vs* Control) as the outcome variable. To quantify the influence of each variable, odds ratios and their confidence intervals were computed, highlighting the most significant factors affecting the diagnosis.

### Statistical analysis

Statistical analyses and data visualisation were performed using GraphPad Prism (version 9.4.0; GraphPad Software, San Diego, CA, USA), SPSS (SPSS statistics version 29.0.0, IBM SPSS Statistics for Windows; IBM, Armonk, NY, USA), and R software (Foundation for Statistical Computing, Vienna, Austria) for the claims data. The probability values of α<0.05 were considered statistically significant.

## Results

### Incidence of probable CPIP and treatment intensity based on healthcare data

**Fig 1:**
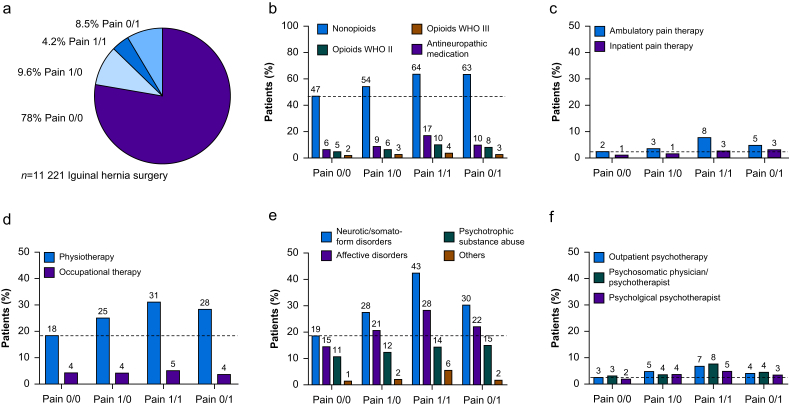
Incidence of ‘probable CPIP’ after hernia surgery and treatment with nonopioids. Patients from a major German health insurance undergoing hernia repair in 2018 were classified by pain before and after hernia surgery using surrogate pain codes from the ICD-10. (a) Percentage of patients categorised in pain 0/0 (*no pain* after surgery); pain 1/0 (only pain before surgery = *pain resolution*); pain 1/1 (pain before and after surgery = *pain persistence*); and pain 0/1 (pain only after surgery = *novel pain*). The latter two are labelled ‘probable CPIP’, because CPIP was not coded in the database. Proportion of patients in ‘probable CPIP’ subgroups receiving nonopioids, antineuropathic medication (anticonvulsants and antidepressants), or weak (WHO II) and strong (WHO III) opioids (b), specialised inpatient and outpatient pain therapy (c), physiotherapy and occupational therapy (d), suffering from psychiatric comorbidities (e), and undergoing psychotherapeutic treatment (f). *n*=11 221. CPIP, chronic postsurgical inguinal pain.

Data from the BARMER health insurance database revealed that 11,221 patients had undergone unilateral inguinal hernia surgery in 2018. As pain was not measured directly, we used pain comorbidities in this anatomical region coded within the ICD-10 1 yr before and after surgery. Patients of the insurance cohort were categorised into four groups: no pain before and after surgery (pain 0/0), pain before but not after surgery (pain 1/0; *pain resolution*), no pain before but pain after surgery (pain 0/1; *novel pain*), and pain before and after surgery (pain 1/1; *pain persistence)* in the subsequent 12 months*.* The latter two were labelled *‘probable CPIP’.* More than three-quarters of the patients did not complain of pain (pain 0/0) (Fig. 1a). Apart from the anatomical repair of the hernia, 9.6% of patients specifically benefited because their presurgical groin pain resolved (pain 1/0). However, an equal number (8.5%) suffered from novel pain (pain 0/1). Males were affected more frequently than females, but persistent pain (pain 1/1) was relatively more common in females.

Prescription of nonopioids for patients with ‘probable CPIP’ was not significantly different from patients without inguinal pain. Treatment with antineuropathic drugs, such as antidepressants and anticonvulsants, was highest in the pain persistence group (2.8-fold) and the novel pain group (1.6-fold) (Fig. 1b). Patients with ‘probable CPIP’ were rarely cared for by a pain specialist, neither as ambulatory (5–8%) nor inpatients (3%) (Fig. 1c). Physical and occupational therapies were also prescribed most frequently in the persistent pain group (Fig. 1d). Psychiatric comorbidities, especially somatoform and affective disorders, were more prevalent but not necessarily more common in patients with ‘probable CPIP’ (Fig. 1e and f). In summary, persistent pain and novel pain after hernia surgery are frequent, but of these patients with ‘probable CPIP’ a maximum of one-fifth receives treatment for chronic pain.

### Sensory phenotyping

**Fig 2:**
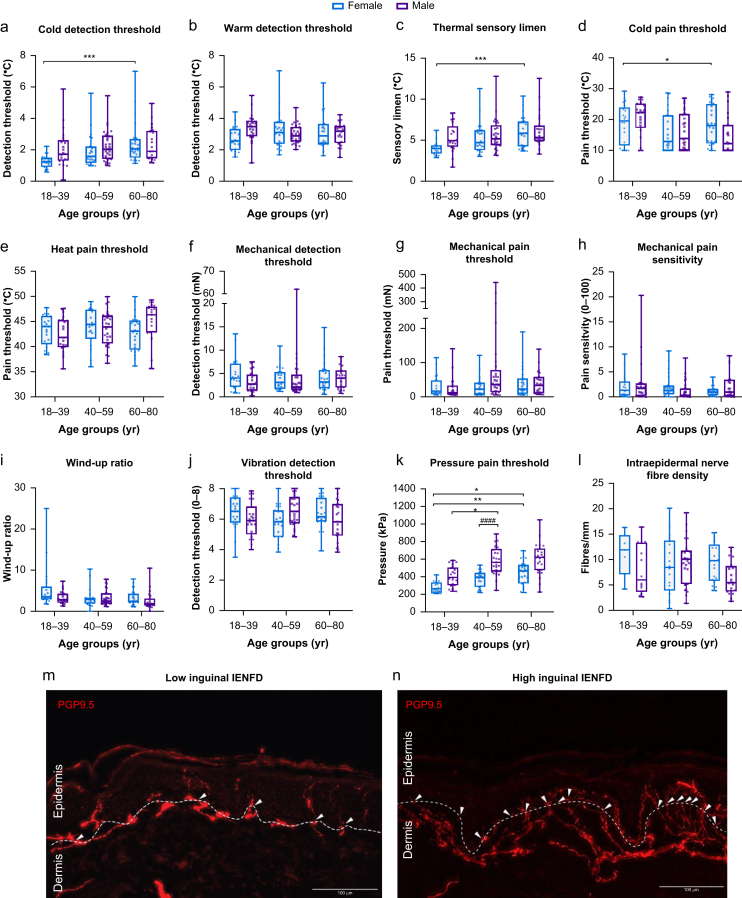
In quantitative sensory testing (QST), pressure pain in the groin was age- and sex-dependent. Healthy controls underwent QST of the groin and skin biopsy. (a) Cold detection threshold; (b) warm detection threshold; (c) thermal sensory limit; (d) cold pain threshold; (e) heat pain threshold; (f) mechanical detection threshold; (g) mechanical pain threshold; (h) mechanical pain sensitivity; (i) wind-up ratio; (j) vibration detection threshold; and (k) pressure pain threshold. (l) Normal values of intraepidermal nerve fibre density (IENFD). Skin punch biopsies were labelled with anti-PGP9.5. Representative examples with few (m) and many (n) fibres depicted. IENFD, intraepidermal nerve fibre density. (a–n) Data are shown by age groups in males and females and presented as median, 25th and 75th percentiles on linear *y*-axes. (a–k) *n*=141; N:n=104. One-way anova on ranks (Kruskal–Wallis test). Star symbols depict significant effects of age within one sex, hashtags show significant gender effects within one age group as follows: ∗^/#^*P*<0.05; ∗∗^/##^*P*<0.01; ∗∗∗^/###^*P*<0.001; ∗∗∗∗^/####^*P*<0.0001.

We deeply phenotyped a selected group of patients of the aforementioned pain cohort. Because of the lack of normative data, we first established age- and sex-dependent reference data for QST^16^ and skin innervation by phenotyping the healthy control group.^17^ Sensory thresholds of the hand were used to ensure that healthy participants (*n*=141) had normal sensation (Supplementary Fig. S2) to be part of the healthy control group.^11^ In the groin, only the deep pressure pain threshold showed a clear dependence on age and sex. Male controls were generally less sensitive and thresholds further increased with age (Fig. 2a–k). Although deep pain thresholds correlated with BMI, the strength was low making variances in subcutaneous fat negligible (Supplementary Table S2).

Free nerve endings in the epidermis are responsible for pain transmission. This innervation is age- and sex-dependent in the distal leg.^17^ In general, groin skin biopsies from healthy controls had a high variance in IENFD with median values of 6–12.5 mm^−2^ (Fig. 2l–n).^18^ Sex and age did not affect IENFD in this region.

### Selective nerve injury and biomarkers in chronic postsurgical inguinal pain

**Fig 3:**
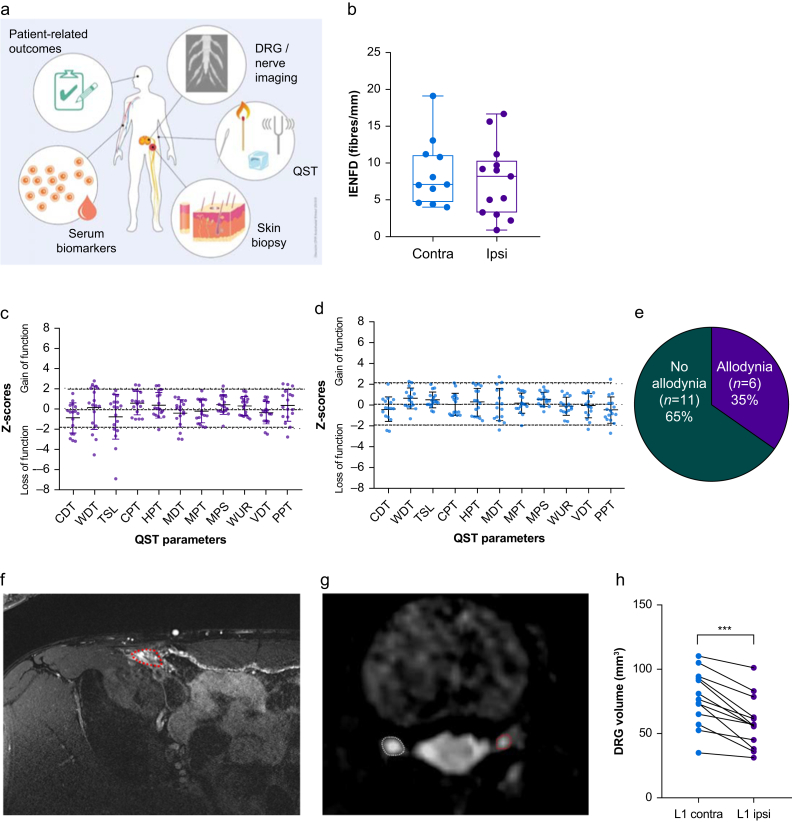
Unaltered sensory profiles and skin innervation in CPIP patients with ipsilateral L1 DRG atrophy. (a) Patients with CPIP from the cross-sectional study underwent deep phenotyping including QST, skin punch biopsies in the groin area, halfway between the iliac spine and the superior ramus of the public bone, and MRI neurography. (b) Sensory fibres were labelled with PGP9.5 to calculate intraepidermal nerve fibre density (IENFD) on the ipsilateral and contralateral sides. Ipsilateral (c) and contralateral (d) sensory thresholds. Dotted lines depict two standard deviations. (e) Fraction of CPIP patients with mechanical allodynia. (f–h) Axial T2-weighted MRI of the groin and bilateral DRG L1 after right-sided hernia repair. (f) Postoperative changes at the level of the inguinal canal (dotted red line) and the adjacent mesh. (g) Comparison of DRG level L1 of a patient with CPIP on the left side (ipsilateral DRG; red) compared with the contralateral unaffected DRG (white). (h) Evaluation of the DRG volumes of the inguinal dermatome of ipsilateral and contralateral groin (L1). Wilcoxon test; ∗∗∗*P*=0.0002 (*n*=17 of these 13 MRI and 11 skin biopsy). CDT, cold detection threshold; CPIP, chronic postsurgical inguinal pain; CPT, cold pain threshold; DRG, dorsal root ganglion; HPT, heat pain threshold; MDT, mechanical detection threshold; MPS, mechanical pain sensitivity; MPT, mechanical pain threshold; QST, quantitative sensory testing; TSL, thermal sensory limit; VDT, vibration detection threshold; WDT, warm detection threshold; WUR, wind-up ratio.

Equipped with normative data, 17 patients with CPIP of the pain cohort were phenotyped to identify biological correlates. All available clinical tests, including patient-reported outcomes, sensory phenotyping, MRI-based imaging, and quantification of serum biomarkers were performed (Fig. 3a).

The pain cohort comprised six females and 11 males. They had undergone surgery 3 months–15 yr before. Although overall age ranged from 21 to 73 yr, females were younger than males at the time of surgery. Other basic characteristics did not differ (Supplementary Table S3). All patients with CPIP experienced moderate mean pain, severe maximum pain, and low disability. Pain had some symptoms of neuropathic origin based on the Neuropathic Pain Symptom Inventory (NPSI). One-third of patients experienced mild symptoms of depression or anxiety. Most patients were taking nonopioids, and only a few were taking antineuropathic medication.

In QST, patients with CPIP had mainly normal thresholds based on Z-values calculated from the healthy controls (Fig. 3c and d). Ten patients reported allodynia (Fig. 3e) or thermal loss. In 11 patients, bilateral skin biopsies were available, 63% had a lower ipsilateral-to-contralateral IENFD ratio (Fig. 3b). Therefore, a subgroup of patients had signs of nerve injury; however, both tests did not necessarily overlap.

On MRIs of the groin and its innervating nerves, inguinal nerves were identified in few patients (Fig. 3f), probably owing to limited spatial resolution and movement artifacts. Potential injuries to the ilioinguinal nerve or ramus genitalis of the genitofemoral nerve could not be excluded. The surgically inserted mesh could be identified medially adjacent to the nerves in only some cases because of movement artifacts. Local inflammatory changes, usually revealed by tissue oedema or contrast agent uptake during i.v. injection as the cause of groin pain, were observed in none of the images. The somas of the ilioinguinal and genitofemoral nerves were located in the bilateral L1 DRG, representing the inguinal dermatome (Fig. 3g). In the voxel-wise MRI DRG morphometry, DRG volumes of the affected side were reduced by 24.4% compared with the healthy contralateral side (*n*=13, Fig. 3h). A reduction in DRG volume was observed in all patients.

**Fig 4:**
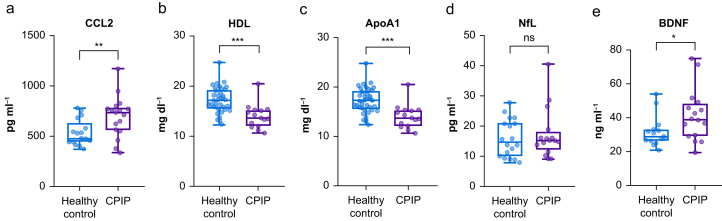
Increase in C-C-motif chemokine ligand (CCL2) and brain-derived neurotrophic factor (BDNF) levels but decrease in HDL and apolipoprotein A1 (ApoA1) levels in chronic postsurgical inguinal pain (CPIP). Nine blood cytokines, chemokines, lipid profiles, and two neuronal markers were measured in patients with CPIP and compared with age- and sex-matched controls. (a) CCL2; (b) HDL; (c) ApoA1; (d) neurofilament light chain (NfL); and (e) BDNF levels in the serum. *n*=15–17, t-test or Whitney–Mann test, with Bonferroni correction for multiple testing. ∗∗*P*<0.05, ∗∗∗*P*<0.001, ns=not significant.

Of the nine cytokines linked to inflammation-induced neuropathic pain syndromes,^9^ only CCL2 was significantly increased in patients with CPIP of the pain cohort compared with sex- and age-matched controls (Fig. 4a). All other cytokines (IL-4, -6, -8, -10, -18, -27, TNF-α, and VEGF) were heterogeneous in patients (Supplementary Fig. S3). Among lipids, total HDL and ApoA1 levels were significantly reduced (Fig. 4b and c), whereas cholesterol, triglycerides, LDL, and ApoA2 levels were unchanged. The BDNF levels were higher in patients with CPIP (Fig. 4e). The axonal damage marker neurofilament light chain (NfL) did not indicate overall group differences (Fig. 4d).

### Dorsal root ganglion atrophy in patients with novel groin pain after surgery

**Fig 5:**
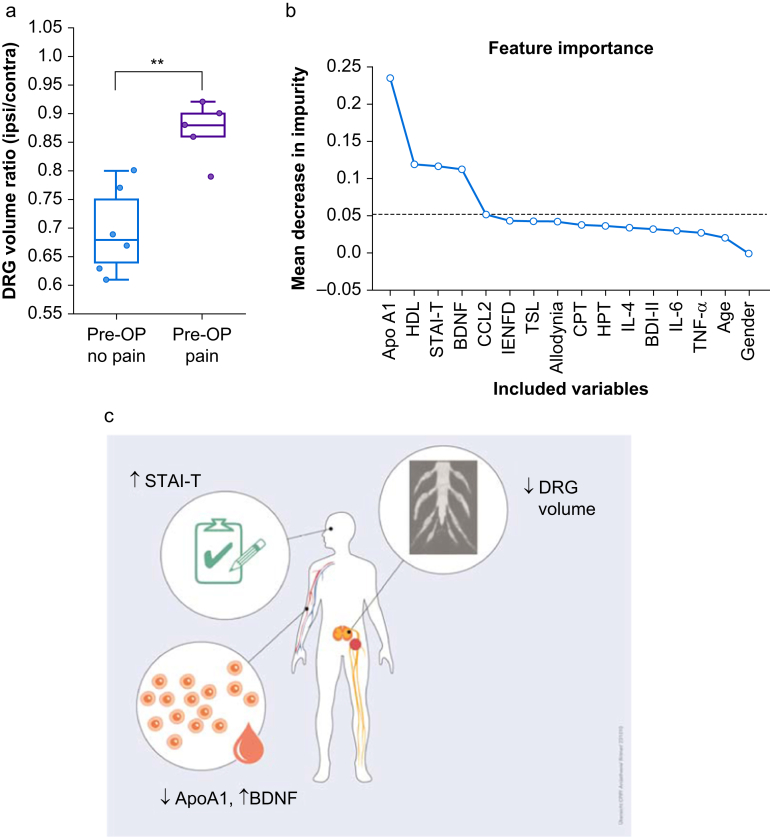
Diagnostic tools and different pathophysiology depending on the presurgical pain. (a) Comparison of the DRG volume level L1 between patients with and without presurgical pain. (b) Mean decrease in impurity was used to compute the most relevant factors. (c) Summary of suggested diagnostic tools. *n*=11, t-test. ∗∗*P*<0.05. ApoA1, apolipoprotein A1; BDNF, brain-derived neurotrophic factor; contra, contralateral; DRG, dorsal root ganglion; ipsi, ipsilateral; Pre-OP, preoperative; STAI-T, State trait anxiety inventory - trait; CCL2, CC-chemokine ligeand 2; IENFD, intraepidermal nerve fibre density; TSL, thermal sensory limen; CPT, cold pain threshold; HPT, heat pain threshold; IL-4, interleukin-4; IL-6. interleukin-6; BDI-II, Beck Depression Index-II; TNF-a, Tumour necrosis factor alpha.

In the insurance cohort, many patients with ‘probable CPIP’ had novel postsurgical pain and did not suffer from pain before the surgery. We therefore subgrouped the data of the pain cohort accordingly. The baseline characteristics did not differ between those with or without presurgical pain (Supplementary Table S4). The ipsilateral L1 DRG volume of patients without presurgical pain was even smaller, pointing towards DRG atrophy, possibly as a result of nerve injury (Fig. 5a).

We further compared the insurance and pain cohort regarding treatment: drug treatment in patients with CPIP of the pain cohort was similar to that in patients with ‘probable CPIP’ of the insurance cohort (nonopioids: 58.8% *vs* 63–64%; opioids: 18% *vs* 11–14%; and antineuropathic drugs: 18% *vs* 10–17% for CPIP *vs* probable CPIP, respectively). Finally, we evaluated whether routine treatment matched with the type of pain (inflammatory *vs* neuropathic); anti-inflammatory drugs were slightly more frequently used by patients with elevated CCL2 levels (60% in patients with high *vs* 42% with low CCL2). Antineuropathic drugs were rarely prescribed (18% of patients), and not more frequently prescribed in patients with allodynia.

### Biomarkers as diagnostic tools

The MDI analysis of the machine learning model revealed that BDNF, ApoA1, CCL2, and STAI-T were the variables with the highest importance (Fig. 5b and c). These variables were subsequently incorporated into a logistic regression model to distinguish CPIP cases of the pain cohort from controls. The logistic regression model demonstrated statistical significance with a log-likelihood *P*-value of <0.001. The model indicated that low levels of ApoA1, along with high levels of BDNF, CCL2, and high scores in the STAI-T, were associated with an increased likelihood of CPIP (Supplementary Table S5).

## Discussion

Annually, 20 million patients worldwide undergo elective hernia repair.^1^ Our study indicates that every 10th patient suffers from persistent pelvic, perineal, and lower abdominal pain or develops novel pain labelled ‘probable CPIP’. This incidence is consistent with the literature.^2^^,^^5^^,^^19^ There was a gap between pain and its proper treatment, for example, interdisciplinary multimodal therapy or antineuropathic medication. Analysis of a separate cohort of patients with CPIP (the ‘pain cohort’) revealed moderate to high pain intensity with low to moderate disability. Deep phenotyping based on the pathophysiology of nerve damage and persistent low-grade inflammation identified ipsilateral DRG atrophy, in combination with low ApoA1, elevated BDNF, and high anxiety levels, as the best additional diagnostic tools. Ipsilateral DRG atrophy is specifically pronounced in patients with novel pain.

Using healthcare claims data, we confirmed that ∼12% of patients of the insurance cohort suffer from ‘probable CPIP’ (pelvic, perineal, and lower abdominal pain) after hernia repair. This is in line with previous prospective^20^ and retrospective data,^5^ but in sharp contrast to other data that reported an incidence of 54%.^2^ Although pain in some patients resolves with hernia repair, 30% of the affected patients (∼80,000 each year worldwide) permanently suffer from it.^20^ Pain impact on daily life is variable. The treatment of ‘probable CPIP’ in our insurance cohort consisted of standard nonopioids after surgery but often lacked antineuropathic medication. In another study, only 2.9% of patients received a new prescription for analgesics.^19^ However, this study underestimated the incidence because it excluded preoperative analgesic prescription, which is a known risk factor. Our patients with ‘probable CPIP’ from the insurance cohort had more psychosocial stress factors. This is in line with previous studies documenting psychological distress, anxiety, catastrophising, reduced pain coping, depression, and hypervigilance as known patient factors for chronic postsurgical pain.^3^^,^^21^ Whether repeat surgery is helpful or even harmful is not completely clear. Stratification based on pain sensitivity to pressure algometry for repeat surgery and neuropathic pain for pharmacotherapy led to a better improvement in the repeat surgery group.^22^ Therefore, there is room for improvement using biomarker-targeted treatments.

One option for markers is sensory testing, which can identify different pathological subtypes in patients with neuropathies of different origins.^16^^,^^23^, ^24^, ^25^ Using healthy controls to generate normative data, we were able to thoroughly map the patients with CPIP in the pain cohort. None of the patients with CPIP had abnormal values in all QST measurements; changes were more subtle than in other diseases.^23^ QST as a biomarker has limitations, not only in CPIP. As a psychophysiological method measuring thresholds, it does not map clinically relevant spontaneous and deep pain in CPIP.^26^, ^27^, ^28^ As we identified allodynia in the clinical examination, which can be easily studied using a brush, QST seems to bring little further diagnostic benefit. Suprathreshold stimulation or new assays for deep fibres will be useful in the future.^26^ Secondly, IENFD was not altered in patients with CPIP of the pain cohort.^25^ Possibly studying nociceptors themselves by labelling transient receptor potential vanilloid 1 (TRPV1) or calcitonin gene-related peptide (CGRP) would be better. In summary, we cannot recommend QST or skin biopsy at this stage.

In our panel, blood concentrations of CCL2, a proinflammatory chemoattractant for monocytes, were increased, as previously seen in preclinical neuropathic pain models^29^^,^^30^ and in patients with polyneuropathy.^9^ In individual patients, TNF-α, IL-4, and IL-6 levels were elevated. Larger groups of patients are needed to better understand which subgroups of patients have elevated cytokines. Among lipids, HDL, with its backbone ApoA1, produced in hepatocytes and known to protect against cardiovascular diseases,^31^ was reduced. Rare mutations of ApoA1 are associated with polyneuropathy (Tangier disease),^32^ and lipids are crucial players in inflammation. For instance, oxidised phospholipids are proalgesic and can be captured by the ApoA1 mimetic D-4F, thus preventing nociceptor activation in acute and chronic inflammatory pain.^33^ The second useful blood biomarker is BDNF, a neurotrophin involved in central sensitisation.^34^^,^^35^ BDNF increases nociceptor excitability in the DRG and the dorsal horn of the spinal cord.^36^ BDNF levels are also increased in fibromyalgia and other primary chronic pain disorders.^37^ In contrast, NfL, a marker of axonal damage, was unchanged in patients with CPIP of the pain cohort.^38^^,^^39^ Thus our data indicate that CPIP has inflammatory and central nervous system remodelling features.

In high-resolution 3D DRG imaging, CPIP was associated with atrophy of the affected L1 DRG in all patients of the pain cohort and was even more pronounced in patients with novel pain after surgery. One possible explanation is the neuronal loss in the outer layer of the DRG, as observed in neuropathy in diabetes mellitus^40^ or plexus injury.^41^ Primary injury to the ilioinguinal or genitofemoral nerve or postsurgical compression or inflammation could be causative.^2^^,^^42^ However, data from preclinical models are controversial whether nerve injury leads to neuronal loss.^43^ In the clinical setting, a recent meta-analysis indicated that ilioinguinal neurectomy is linked to lower incidence of CPIP.^44^ More prospective data are needed to solve this. Direct detection of peripheral nerve injury of the genitofemoral or ilioinguinal nerve on MRI was not possible because of motion artifacts and limited spatial resolution. Further DRG imaging techniques such as microstructural or quantitative functional MRI will be valuable in the future.

To test the predictive power of deep phenotyping, in addition to DRG atrophy, an AI-based model was developed that included all measures. After excluding irrelevant variables, ApoA1, BDNF, and STAI-T were identified as the best predictors and biomarkers for CPIP. Lower levels of ApoA1 and higher levels of BDNF and STAI-T were associated with a higher probability of CPIP in our sample. Although the latter serum values and questionnaires are relatively easy to obtain, MRI can be obtained on routine scanners but will need analyses by experienced personnel. However, our model is not intended as a substitute for clinical diagnosis. Furthermore, validation in future patient cohorts will be important.

A major limitation of healthcare data is the lack of an ICD-10 code for CPIP and no repeated pain measurements. Therefore, we cannot exclude that other undiagnosed painful gynaecological, urological, or orthopaedic conditions might have been the cause of pain. Claims data are designed for reimbursement and might therefore be biased. Secondly, we did not know the cause of analgesic prescriptions, such as inguinal pain or other diseases. Our cross-sectional clinical study was limited by the small number of participants.

In summary, we provide data indicating that many patients who undergo elective hernia surgery suffer from new-onset groin pain. Therefore, patients undergoing hernia surgery should be informed of this potential complication. Based on our exploratory study, we provide clinicians with additional diagnostic tools for CPIP, including DRG MRI, ApoA1, and BDNF serum levels, and anxiety screening with the STAI test. Together, our findings suggest that individual therapies can be targeted based on a core set of biomarkers.

## Authors’ contributions

Conception and design: GK, RB, WM, AB, MP, CS, NS, HLR

Conception and design of claims data: JD, DS

Acquisition, analysis, and interpretation of clinical and QST data: EH

Acquisition, analysis, and interpretation of MRI data: MSc

Acquisition, analysis, and interpretation of claims data: GK, FA, NR, KT

Acquisition, analysis, and interpretation of blood biomarker data: A-KR, NC

Analysis and interpretation of patient data: MM, AW

Acquisition, analysis, and interpretation of skin data: EH, YJ

Statistical analysis and interpretation of data: AS

Analysis and interpretation of biomarker data: MSe

Manuscript drafting: EH, MSc, GK, A-KR, NC, FA, NR, KT, JD, DS, MM, AW, YJ, AS, MSe

Critical revision of the manuscript: RB, WM, AB, MP, CS, NS, HLR

Final approval: all authors

## Declarations of interests

HR received consultant fees from Gruenenthal and Orion, and financial support for a study by Algiax. WM reports grants from Gruenenthal and Pfizer, personal fees from Tafalgie, Kyowa, Mundipharma, Grünenthal, Ethypharm, and Spectrum Therapeutics. CS has received consulting fees from Algiax, Bayer, Grifols, Immunic, Merz, Roche, and Takeda Pharmaceuticals, and has given educational talks for Teva, CSL Behring, Grifols, GlaxoSmithKline, Takeda Pharmaceuticals, Pfizer, Amicus, and Alnylam. All other authors have no conflict of interest regarding this work.
