# Supplementary material for: Chronic postsurgical inguinal pain: incidence and diagnostic biomarkers from a large German national claims database
Source: Br J Anaesth. 2025 Feb 4;134(6):1746–55. doi: 10.1016/j.bja.2024.11.048 (PMC12106889; doi:10.1016/j.bja.2024.11.048)
Supplement: Multimedia component 1 [file mmc1.pdf]

Supplementary information

Materials and Methods

Analysis of the health care data for the insurance cohort

We used anonymized nationwide administrative claims data warehouse for inpatient and outpatient treatment as well as core data of a German administrative health care claims data (BARMER GEK). In Germany, the vast majority of workers, along with their families, are covered by public health insurances. Public insurances provide comprehensive coverage, including hospital and outpatient care, medications, and other therapeutic aids. 90% of the population in Germany are covered by public insurances. Thus, these data are quite reflective of the broader German demographic. Various public health insurance schemes exist, such as BARMER, offering similar benefits and collecting comparable data. Individuals are free to choose among different public health care plans. Minor variations exist among different insurance plan members due to historical factors; for example, BARMER's enrollees include a higher proportion of women, yet it still offers a good representation of Germany's population demographics (approximately 9 Mio insured persons, 10% of total German population).[1] In terms of medications, the German claims database records every prescription issued by doctors for outpatient care under each health insurance scheme, but lacks data on in-hospital prescriptions. In summary, we accessed outpatient opioid prescriptions from Germany's second-largest health insurer, providing a significant representation of the national populace.

The study was approved by the ethics committee of the Jena University Hospital and the data protection officer of the German federal state of Thuringia (the German Clinical Trials Register DRKS00024588). Since claims data were anonymous, the need for informed consent was waived. The description of the study follows the Reporting of studies Conducted using Observational Routinely-collected Data (RECORD) guidelines.[2]

Using the Operationen- und Prozedurenschlüssel (OPS) classification (OPS codes 5-530 or 5-531), which is the German modification of the international classification of procedures in medicine, we identified cases who underwent hernia surgery in 2018. Since CPIP was not coded in the ICD-10, surrogate diagnoses like R10-4 (pelvic and perineum pain; pain with localization in other parts of the lower abdomen; other and unspecified abdominal pain) or M79.25 (Neuralgia and neuritis, unspecified: Pelvic region/thigh) were used. Sociodemographic and health care parameters, such as pain medication, outpatient and multimodal pain therapy, psychiatric comorbidities, physical and occupational therapy were obtained one year before and after the index year 2018.

Patient and control cohorts

The cross-sectional unicenter *ResolvePAIN* study protocol was registered at the German registry for clinical studies (https://www.germanctr.de/) (Registration Number DRKS00016790). Study participants for the pain cohort were recruited from the outpatient clinic of the Center for Interdisciplinary Pain Medicine or approached during a follow-up study at the Dept of Surgery.[3] Patients were ≥18 y, of both sexes and gave written informed consent. Ethical approval was obtained from the responsible ethics committees at the study centers. Pain of other origin at the side of investigation, current or past malignancies, surgeries that took place within the past 4 weeks, autoimmune, neurological, and major psychiatric diseases as well as diabetes were exclusion criteria. Seventeen patients were included, 11 males and 6 females (**Table 1**).

The healthy control group of the *ResolvePAIN* study was recruited using newspaper ads and comprised 141 cases based on a sample size calculation using variability of pressure pain thresholds based on age and sex.[4] In addition to the criteria mentioned above, subjects with any type of previous inguinal surgery were excluded from the control group. The controls were subdived by three age groups (**Supplementary Table 1**).

Clinical assessment and patient reported outcomes

The patient's history included age, education, employment status, medical and drug history as well as family history for chronic pain, psychiatric and neurological diseases. Last menstrual period or if applicable menopausal state were assessed in females. Basic clinical parameters were measured for each participant (weight, height, body mass index). All study participants underwent a clinical neurological examination and quantitative sensory testing.

In addition, patients filled in standardized patient reported outcomes[5]: The neuropathic pain inventory (NPSI), German version, (range 0-100) describes the expression of different neuropathic pain characteristics. It was designed to evaluate the different symptoms of neuropathic pain, to differentiate between subtypes of neuropathic pain and to possibly verify whether symptoms respond differentially to various pharmacological agents or other therapeutic interventions. The Graded Chronic Pain Scale, German version, (GCPS) classifies into four chronic pain grades (Grade I – IV) based on pain intensity items and disability items. Depression was measured by the Beck Depression Inventory 2, German version, (BDI-II, range 0-63). BDI-II results between 0 and 13 are considered minimal depression, 14-19 mild depression, 20-28 moderate depression and 29-63 severe depression. The State-Trait Anxiety Inventory, German Version, (trait anxiety subscale STAI-T, range 20-80) was filled in to record anxiety symptoms. For the STAI-T, a value </= 39 was defined as normal.

Quantitative sensory testing (QST)

All healthy controls and CPIP patients underwent QST following the standardized DFNS (German Research Network on Neuropathic Pain) protocol.[6] All measurements were performed by a formally trained investigator. The affected inguinal side was defined as the test and the contralateral side as control side. Measurements were conducted halfway between spina iliaca anterior superior and ramus superior of the pubic bone. In control subjects, the thenar region of the hand was assessed first before moving on to the groin.

QST consisted of 11 single tests resulting in 13 parameters, subsequently applied to the selected area beginning with the control side. It includes determination of temperature (cold detection threshold, CDT; warm detection threshold, WDT, thermal sensory limen, TSL, i.e. thermodiscriminative function for alternating cold and warm stimuli) and thermal pain (pain cold pain threshold, CPT; heat pain threshold, HPT) thresholds. Paradoxical heat sensations (i.e., cold stimulation is perceived as heat pain, PHS) were counted if present. Next, mechanical detection threshold (MDT), mechanical pain threshold (MPT), mechanical pain sensitivity (MPS), dynamic mechanical allodynia (DMA), the wind-up ratio for painful stimuli (WUR), the vibration detection threshold (VDT) and the pressure pain threshold (PPT) were determined.

Data from healthy controls were first used to generate normal values grouped by age and sex. Values from CPIP patients were then analyzed after z-transformation. To allow for log-transformation the constant 0.1 was added to the mean value which prevented measurements with a mean of zero from being excluded. After log-transformation the following equation was used to obtain z-values:

$$z = \frac{(value\ of\ the\ subject) - (mean\ of\ healthy\ control\ group)}{SD\ of\ healthy\ control\ group}$$

According to Magerl et al., raw data were used for z-score computation in CPT, WDT and VDT, while other parameters were used in logarithmic form. Z-Scores were calculated with each age- and gender-based control group being used to determine mean and standard deviation needed.[4, 7]

Healthy controls were subdivided into groups according to gender (female, male) and age (18-39 y, 40-59 y, 60-80 y). QST measurements of control subjects' thenar regions were referenced to data previously published by the DFNS (**Supplementary Figure 2**).

Standardized skin biopsies and immunohistochemistry

Skin biopsies were aseptically obtained using a 5 mm punch after local application of mepivacaine. Due to the small size of the punch, sutures were not needed. The biopsies were performed at the same location as the QST measurements. Bilateral biopsies were obtained from patients and unilateral ones from controls. Each biopsy was divided into two pieces: one part was used for immunohistochemistry, one shock frozen for RNA analysis.

Skin biopsy specimens were fixed in 4% paraformaldehyde for 30 min, then subsequently washed in PBS in three 10 minute-intervals before overnight storage in 10% sucrose until freezing and embedding in OCT. [7, 8] Samples were cut into 50 µm thickness and mounted on microscope slides. The slides were dried at room temperature (RT) for 30 min, outlined with a hydrophobic barrier using Dako Pen, and blocked with 10% BSA for 30 min at RT in a humid chamber. A combination of the primary antibody against PGP9.5 (Zytomed systems, 516-3344, Berlin, Germany, 1:200) overnight at 4°C in a humid chamber and the secondary antibody Cy3 (Jackson ImmunoResearch, West Grove, PA, USA, 1:50) 2 hours at RT before mounting in Vectashield containing DAPI and final storage at 4°C. Nerve fibers were counted according to guideline of the European Federation of Neurological Societies.[8]

Molecular assessments, standardized skin biopsies and immunohistochemistry

Serum blood was drawn from all patients with CPIP and 17 age- and sex-matched healthy controls in the morning after a 6 h fasting period (S-monovette white, 7.5 ml, Sarstedt). Cytokines were analyzed using predesigned assays (9-plex with IL-4, -6, -8, -10, -18, -27, TNFα, VEGF, and CCL2; Merck Millipore) selected from previous studies on neuropathic pain and CPIP.[9] Analyses were performed in duplicate with Luminex®100 (Luminex Corporation, Austin, Texas, USA). Neurofilament light chain (NfL) and brain-derived neurotrophic factor (BDNF) levels were measured using the ELLA technique.[10, 11] Lipids (cholesterol, high-density lipoprotein (HDL), low-density lipoprotein (LDL), triglycerides, apolipoprotein A1 (ApoA1), and A2 (ApoA2) were measured at the hospital's main laboratory. Specifically, the cobas lipid panel and APOAT assay (Roche Diagnostic, Vienna, Austria) and thus homogenous enzymatic colorimetric and immunoturbidimetric testing were employed.[12-14] If patients or controls consented, both groups underwent bilateral groin skin punch biopsies (5 mm) to quantify the IENFD marked by PGP9.5. Four patients and 37 controls did not consent to skin biopsy.

Imaging protocol and analysis

All MRI DRG examinations of CPIP patients were conducted on the same MRI 3 Tesla scanner between October 2018 and June 2022 (Magnetom PRISMAfit Siemens Healthineers, Erlangen, Germany)[15] using a high-resolution three-dimensional T2-w FSE sequence (SPACE: sampling perfection with application optimized contrasts using different flip angle evolution) with spectral fat saturation of the lumbosacral plexus and spine (scanning parameters: FOV 300 × 295 × 106 mm^3, voxel size 1.1 × 1.1 × 1.1 mm^3, ΔTE:4.4 ms, TE:301 ms, TR:2000 ms). The imaging slab was aligned perpendicular to the L1 vertebral body. DRGs were analyzed using 3D Slicer.[16] the volumes of bilateral DRG at lumbar level L1 were determined by voxel-based volumetric analysis for 26 DRGs by two blinded experts with over five years of neuroradiological experience (n = 13). Four patients did not consent to the DRG MRI.

Building a prediction model for CPIP

A predictive model was developed to anticipate the presence of CPIP using objective measures. The model was tested on a sample of 17 CPIP patients and 17 age- and sex-matched healthy controls. Predictor variables included various lab values, QST values, STAI-T score, BDI score, age, and gender.

The model construction followed a two-step approach: first, identifying relevant variables using a random forest algorithm, and second, building the prediction model using logistic regression. Random forest is a machine learning approach used for classification and regression. Aside from its ability to provide accurate predictions,[17] RF also provides measures for variable importance.[18] One of these

measures is the mean decrease in impurity (MDI), which measures the relevance of each variable to the categorization task, where higher MDI values are associated with higher importance.

Through MDI analysis, the most important five variables identified were ApoA1, HDL, STAI-T, BDNF, and CCL2. Due to high correlation with Apo A1, HDL was excluded, ensuring no multicollinearity (VIF < 2.5) among the remaining variables. These selected variables were then entered into a binary logistic regression model to predict the diagnosis (CPIP vs. healthy control).

The random forest's MDI was computed using the python package 'scikit-learn'[19], and the logistic regression model was built using the python package 'statsmodels'.[20]

Statistical analysis [19]

Normal distribution for groin measurements was assessed using the Shapiro-Wilk-Test, but only met for HPT without logarithmic transformation. MDT and MPS were normally distributed after log transformation. As not all parameters were normally distributed despite log transformation, the Kruskal–Wallis test was used to determine differences between the control groups. Spearman's rho was used to determine the association between the measurements and clinical data. Mean values for DRG volumes of ipsilateral affected and contralateral healthy sites were calculated and tested for statistical significance using the Wilcoxon matched-pair signed-rank test.

Supplementary tables

Controls			
**Characteristics**	**All (n = 141)**	**Females (n = 68)**	**Males (n = 73)**
Age groups			
18-39 y (n)	42	21	21
40-59 y (n)	52	23	29
60-80 y (n)	47	24	23
BMI [kg/m^2] median (range)	24.1 (19.1-37.4)	23.7 (19.7-34.3)	24.3 (20.2-37.4)
BDI-II [0-63] median (range)	2.0 (0 – 24)	2.0 (0 – 24)	2.5 (0 – 19)
STAI-T [20-80] median (range)	31.0 (20-54)	31.0 (20-54)	32.0 (20-53)

Supplementary Table 1: Clinical characteristics of the healthy control group comparing males and females. BMI, body mass index; BDI-II, Beck Depression Index-II; STAI-T, State-Trait Anxiety Inventory.

QST Parameter	ρ	p-value
**CDT**	0.241	0.004
**WDT**	0.219	0.010
**TSL**	0.259	0.002
**CPT**	-0.044	0.607
**HPT**	0.034	0.696
**MDT**	0.198	0.020
**MPT**	0.193	0.023
**MPS**	-0.160	0.060
**WUR**	0.45	0.609
**VDT**	-0.182	0.032
**PPT**	0.162	0.058

Supplementary Table 2: Spearman's correlation between BMI and QST parameters. BMI, body mass index; QST, quantitative sensory testing; CDT, cold detection threshold; WDT, warm detection threshold; TSL, thermal sensory limen; HPT, heat pain threshold; CPT, cold pain threshold; MDT, mechanical detection threshold; MPS, mechanical pain sensitivity; MPT, mechanical pain threshold; WUR, wind-up ratio; VDT, vibration detection threshold; PPT, pain pressure threshold.

**Characteristics**	**CPIP (n = 17)**	**Healthy controls (n = 17)**
Age at inclusion [years] median (range)	42 (21 - 73)	52 (23 - 69)
Age at operation [years] median (range)	40 (21 - 70)	-
Years since surgery median (range)	2 (0 - 6)	-
BMI [kg/m^2] median (range)	24 (17 - 30)	24 (22 - 37)
**Type of surgery (number)**		
Open/TEP/TAP/not specified	6/5/3/3	-
**Pain & mental health assessment**		
Mean pain [0-10] median (range)	4 (2 - 5)	-
Min. pain [0-10] median (range)	2 (0 - 6)	-
Max. pain [0-10] median (range)	6 (3 - 10)	-
NPSI [0-100] median (range)	16 (0 - 61)	-
**GCPS [I-IV]** (n)		
**I/II/III/IV**	9/5/0/0	-
BDI-II [0-63] median (range)	5 (0 - 19)	2 (0 - 11)
BDI-II participants over cut-off mild depression (n)	6	1
STAI-T [20-80] median (range)	36 (20 - 48)	32 (23 - 40)
STAI-T participants over cut-off for anxiety (n)	4	0
**Treatment (n-number)**		
Non-Opioids/Opioids/Antineuropathics	10/3/3	-
Local treatment	2	-
Surgical neurolysis	0	-
Physiotherapy	5	-

Supplementary Table 3: Clinical characteristics of CPIP patients and age- and sex-matched healthy controls. BMI, body mass index; TEP, total extraperitoneal patch plasty; TAPP, transabdominal preperitoneal patch plasty; NPSI, Neuropathic Pain Symptom Inventory; GCPS, Graded Chronic Pain Scale; BDI-II, Beck's Depression Index-II; STAI-T, State-Trait Anxiety Inventory-Trait. In case numbers did not add up to 17, items were not available for all patients.

CPIP patients		
**Characteristics**	**Pain persistence (n = 7)**	**Novel pain (n = 7)**
Age at inclusion [years] median (range)	42 (33 - 67)	48 (25 - 73)
Age at operation [years] median (range)	40 (30 - 62)	50 (23 - 70)
Years since surgery median (range)	3 (1 - 5)	2 (1 - 6)
BMI [kg/m^2] median (range)	24 (21 - 28)	23 (18 - 30)
**Type of surgery (number)**		
Open/TEP/TAP/not specified	4/2/1/0	2/2/1/2
**Pain & mental health assessment**		
Mean pain [0-10] median (range)	4 (4 - 5)	3 (2 - 5)
Min. pain [0-10] median (range)	2 (1 - 6)	2 (0 - 5)
Max. pain [0-10] median (range)	8 (4 - 10)	6 (3 - 10)
NPSI [0-100] median (range)	20 (2 - 32)	14 (0 - 61)
**GCPS [I-IV]** (n)		
**I/II/III/IV**	3/4/0/0	4/1/0/0
BDI-II [0-63] median (range)	8 (0 - 18)	2 (0 - 19)
BDI-II patients over cut-off mild depression (n)	3	2
STAI-T [20-80] median (range)	36 (20 - 48)	30 (26 - 39)
STAI-T patients over cut-off for anxiety (n)	2	0
**Treatment (n-number)**		
Non-Opioids/Opioids/Antineuropathics	4/1/2	3/1/0
Local treatment	1	0
Surgical neurolysis	0	0
Physiotherapy	2	3

Supplementary Table 4: Clinical characteristics of CPIP patients comparing with pain persistence (pain pre surgery) and new pain (no pain before surgery). BMI, body mass index; TEP, total extraperitoneal patch plasty; TAPP, transabdominal preperitoneal patch plasty; NPSI, Neuropathic Pain Symptom Inventory; GCPS, Graded Chronic Pain Scale; BDI-II, Beck's Depression Index-II; STAI-T, State-Trait Anxiety Inventory-Trait. In case numbers did not add up to 14, items were not available for all patients.

	OR (95% CI)	p-value
**Constant**	1.34 (0.47 - 3.88)	0.58
**BDNF**	3.74 (0.7 - 20.07)	0.12
**ApoA1**	0.23 (0.06 - 0.92)	0.04
**CCL2**	1.7 (0.5 - 5.75)	0.4
**STAI-T**	1.66 (0.5 - 5.47)	0.4

Supplementary Table 5: Binary logistic regression analysis for the variables predictive of the presence of CPIP. STAI-T = state trait anxiety inventory – trait. BDNF = brain derived neurotrophic factor. ApoA1 = apolipoprotein A1.

Supplementary Figures

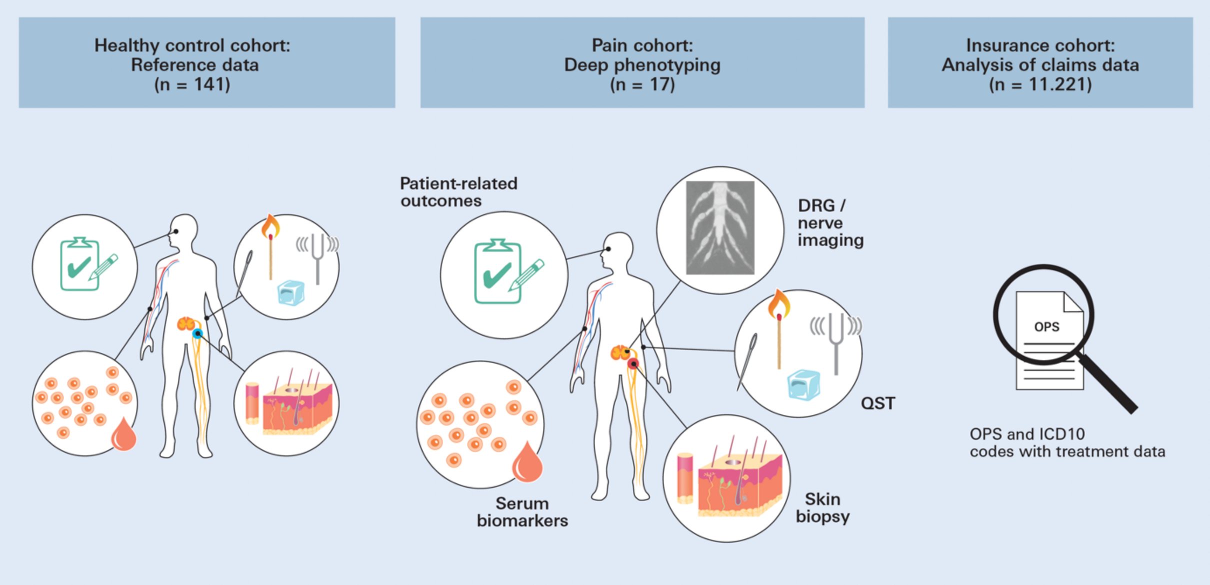

Supplementary Figure 1: Overview of testing for the healthy control, pain, and insurance cohort. The healthy control cohort (left) underwent Quantitative Sensory Testing (QST) to create reference values for the groin. Additionally, they filled out questionnaires and blood was drawn. If consent was given, an inguinal skin biopsy was performed. The pain cohort (middle) underwent the same battery of testing with the addition of an MRI of the L1 dorsal root ganglion. In the insurance cohort (right), claims data who underwent groin hernia repair in 2018 were analysed.

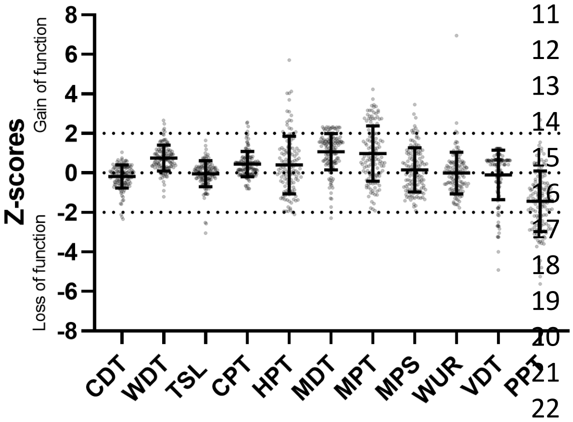

Supplementary Figure 2: Control quantitative sensory testing values of the right hand in healthy controls. Cold detection threshold (CDT), warm detection threshold (WDT), thermal sensory limen TSL), cold pain threshold (CPT), heat pain threshold (HPT), mechanical detection threshold (MDT), mechanical pain threshold (MPT), mechanical pain sensitivity (MPT), wind-up ratio (WUR), vibration detection threshold (VDT), and pressure pain threshold (PPT) are displayed. N = 141 healthy controls. Dotted lines delineate the range of two standard deviations.

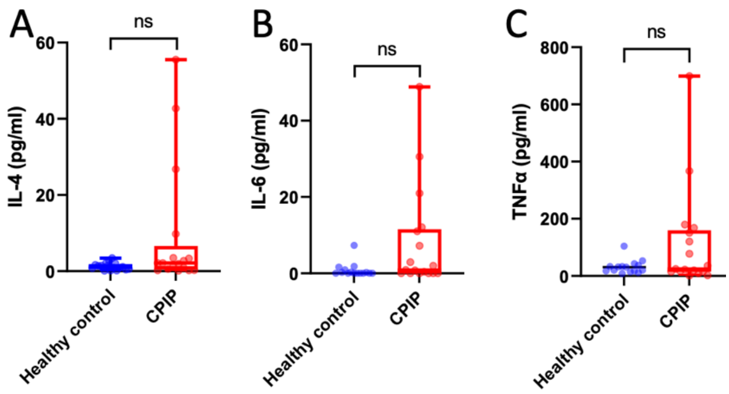

Supplementary Figure 3: No difference in IL-4, IL-6, and TNFα. Blood cytokines were measured in CPIP patients compared to age- and sex-matched controls. **A** Interleukin (IL)-4 **B** IL-6 **C** Tumor necrosis factor (TNF)α. n = 15-17, t-test or Whitney-Mann test, with Bonferroni correction for multiple testing. ns = not significant.

References

1 Marschall U, L'Hoest H, Radbruch L, Hauser W. Long-term opioid therapy for chronic non-cancer pain in Germany. *Eur J Pain* 2016; **20**: 767-76

2 Benchimol EI, Smeeth L, Guttmann A, et al. The REporting of studies Conducted using Observational Routinely-collected health Data (RECORD) statement. *PLoS Med* 2015; **12**: e1001885

3 Widder A, Reese L, Lock JF, et al. Postoperative Analgesics Score as a Predictor of Chronic Postoperative Inguinal Pain After Inguinal Hernia Repair: Lessons Learned From a Retrospective Analysis. *World J Surg* 2023; **47**: 2436-43

4 Magerl W, Krumova EK, Baron R, Tolle T, Treede RD, Maier C. Reference data for quantitative sensory testing (QST): refined stratification for age and a novel method for statistical comparison of group data. *Pain* 2010; **151**: 598-605

5 Dietz C, Muller M, Reinhold AK, et al. What is normal trauma healing and what is complex regional pain syndrome I? An analysis of clinical and experimental biomarkers. *Pain* 2019; **160**: 2278-89

6 Rolke R, Magerl W, Campbell KA, et al. Quantitative sensory testing: a comprehensive protocol for clinical trials. *Eur J Pain* 2006; **10**: 77-88

7 Hartmannsberger B, Doppler K, Stauber J, et al. Intraepidermal nerve fibre density as biomarker in Charcot-Marie-Tooth disease type 1A. *Brain Commun* 2020; **2**: fcaa012

8 Lauria G, Cornblath DR, Johansson O, et al. EFNS guidelines on the use of skin biopsy in the diagnosis of peripheral neuropathy. *Eur J Neurol* 2005; **12**: 747-58

9 Garcia-Fernandez P, Hofflin K, Rausch A, et al. Systemic inflammatory markers in patients with polyneuropathies. *Front Immunol* 2023; **14**: 1067714

10 Cebulla N, Schirmer D, Runau E, et al. Neurofilament light chain levels indicate acute axonal damage under bortezomib treatment. *J Neurol* 2023; **270**: 2997-3007

11 Andreska T, Rauskolb S, Schukraft N, et al. Induction of BDNF Expression in Layer II/III and Layer V Neurons of the Motor Cortex Is Essential for Motor Learning. *J Neurosci* 2020; **40**: 6289-308

12 Siedel J, Schiefer S, Rosseneu M. Immunoturbidimetric Methodfor Routine Determinations of Apolipoproteins A-I, A-II and B in Normo-and Hyperlipemic Sera compared with Immunonephelometry. *ClinChem* 1988; **34**: 1821-5

13 Siedel J, Schmuck R, Staepels J. Long term stable, liquid ready-to-use monoreagent for the enzymatic assay of serum or plasmatriglycerides (GPO-PAP method). AACC Meeting Abstract 34. . *ClinChem* 1993 1127.

14 Katayama Y, H. S, Fujinaka M. Evaluation of New Homogeneous Assay Kit to Determine HDL-C with a High Reactivity with Cholesterol in Various Types of HDL. *AACC Meeting* 2009: Poster Abstract B-103

15 Weiner S, Strinitz M, Herfurth J, et al. Dorsal Root Ganglion Volumetry by MR Gangliography. *AJNR Am J Neuroradiol* 2022; **43**: 769-75

16 Fedorov A, Beichel R, Kalpathy-Cramer J, et al. 3D Slicer as an image computing platform for the Quantitative Imaging Network. *Magn Reson Imaging* 2012; **30**: 1323-41

17 Gao D, Chen Q, Zeng Y, Jiang M, Zhang Y. Applications of Machine Learning in Drug Target Discovery. *Curr Drug Metab* 2020; **21**: 790-803

18 Breiman L. Manual on setting up, using, and understanding random forests v3. 1. . *Statistics Department University of California Berkeley, CA, USA* 2002

19 Pedregosa F, Varoquaux G, Gramfort A, et al. Scikit-learn: Machine learning in Python. *J Machine Learning Res* 2011; **12**: 2825-30

20 Seabold S, Perktold J. Statsmodels: Econometric and Modeling with Python. Conference,. *9th Python in Science, Austin, 28 June-3 July, 2010,* 2010: A57-61
